# Diet quality indices and gastrointestinal cancer risk: results from the Lifelines study

**DOI:** 10.1007/s00394-021-02648-3

**Published:** 2021-08-02

**Authors:** Sara Moazzen, Francisco O. Cortes-Ibañez, Bert van der Vegt, Behrooz Z. Alizadeh, Geertruida H. de Bock

**Affiliations:** 1 Department of Epidemiology, FA40 University Medical Centre Groningen, University of Groningen, PO 30.001, Groningen, 9700 RB The Netherlands; 2 Molecular Epidemiology Research Group, MDC Berlin-Buch, Max-Delbrück-Center for Molecular Medicine in der Helmholtz-Gemeinschaft, Berlin, Germany; 3 Department of Pathology and Medical Biology, University of Groningen, University Medical Center Groningen, Groningen, the Netherlands

**Keywords:** Gastrointestinal neoplasms, Oesophageal neoplasms, Stomach neoplasms, Colorectal neoplasms, Nutrition quality

## Abstract

**Objective:**

To investigate the long-term association between four dietary quality indices and the risk of gastrointestinal (GI) cancer.

**Methods:**

Baseline details of the dietary intake of participants, assessed by a single food frequency questionnaire from the prospective Lifelines population-based cohort were translated to diet quality scores using several dietary and dietary-lifestyle indices. Incident cases of GI cancer were then assessed by linkage to the Dutch nationwide histo-cytopathology registry. The association between GI cancer risk and diet quality (defined as higher quintiles on dietary indices compared to the first quintile) was assessed by multivariable Cox proportional hazard models.

**Results:**

We included 72,695 participants aged 51.20 ± 8.71 years with a median follow-up to cancer diagnosis of 8 years (interquartile range 2 years). During follow-up, 434 colorectal cancers and 139 upper GI cancers were diagnosed. There was a significant reduction in colorectal cancer risk for high categories in the American Cancer Society (ACS) Index (hazard ratio 0.62; 95% CI 0.46–0.84). However, high dietary index scores were not associated with strong beneficial effects on upper GI cancer risk.

**Conclusion:**

High quintiles on the ACS Index were associated with a significantly reduced risk of colorectal cancer. This index may be of use in a colorectal cancer prevention program.

**Supplementary Information:**

The online version contains supplementary material available at 10.1007/s00394-021-02648-3.

## Introduction:

Gastrointestinal (GI) cancers are the most common cancer worldwide, having an annual incidence of approximately 4.5 million cases [1–3]. Evidence of the benefits of modifying dietary and physical activity on preventing chronic disease, including GI cancer, has led to the formulation of multiple dietary guidelines. In turn, various indices have been developed to quantify adherence to these guidelines, including general diet quality indices (based on evidence for dietary elements involved in preventing chronic disease), and diet-related lifestyle indices (these include food components, physical activity, and adiposity measures). It is thought that better adherence to diet quality indices will help to improve cancer prevention [4, 5].

The benefits of high diet quality in the prevention of GI cancer have not been shown consistently across cancers of the lower and upper GI tract. Some studies have shown that high dietary quality is unrelated to a lower risk of developing colorectal cancer (CRC) [2, 3, 6] or upper GI cancers [2, 7–9]. In contrast, findings from other perspective investigations have reported a significantly reduced risk for upper GI tract cancers and CRC among individuals with high-quality diet defined by diet quality indices [9–13]. Similarly, our recent meta-analyses of related studies revealed 1.2–1.5-times reduced risk for CRC [14] and an approximate 1.4–1.7-times reduced risk for upper GI cancers [15], in individuals with higher diet quality. These inconsistencies arise from differences in the indices for measuring diet quality across studies, the follow-up duration, and the study population [14, 15]. Moreover, the role of high diet quality, defined by diet-related lifestyle indices, is also inconclusive concerning the impact on the risk of GI cancer. Whereas some research has shown a 1.36–2.08 times reduction in the risk of CRC among individuals with high diet-related lifestyle indices [16–18], a recent study has failed to confirm these findings [19]. In addition to the variations in the studies populations, the finding have been disputed due to differences in the applied indices, applying self-report questionnaires for determining cases of GI cancer, possibility misclassification of outcomes, and having too few incident cases, resulting in a low power to detect an association [19].

It is crucial to improve our assessment of the association between diet quality, as quantified by various diet quality indices, and the risk of GI cancer. This should be performed in a large-scale homogenous population where both the outcome of occurrence of GI cancer is measured objectively by validated pathological reports and the diet indices are measured and scaled using the same harmonized and standardized questionnaire. In this study, we, therefore, investigated the association between diet quality, as quantified by generally used dietary indices and cancer-specific diet indices, and the incidence of GI cancer in a general population of Dutch adults who participated in the prospective Lifelines cohort. To give a comparison between the performance of generally used dietary indices and cancer-specific diet indices in prevention of GI cancers, we quantified the diet quality of study participants with generally used dietary indices and cancer-specific diet indices. Among frequently used general diet quality indices, due to the lack of information on micronutrients and macronutrients intake in the Lifelines database, we applied food-based general diet quality indices to quantify diet quality among the study population.

## Materials and method

### Study design and population

We studied a subgroup of 72,695 adults (age ≥ 40 years) included in the Dutch Lifelines cohort between 2006 and 2009 [20]. Lifelines is a prospective, multi-disciplinary, population-based cohort study of 167,729 individuals, and it has a major focus on multi-morbidity and complex genetics, applying a wide variety of investigative procedures to assess the sociodemographic, biomedical, behavioral, psychological, and physical factors crucial to health and disease in the general population. Participants were recruited by general practitioners, and family members were subsequently invited to participate, but adults could also self-register. Lifelines are conducted according to the principles of the Declaration of Helsinki and were approved by the medical ethics committee of the University Medical Center Groningen (the Netherlands). Written informed consent was obtained from all participants for the Lifelines study.

**Fig. 1:**
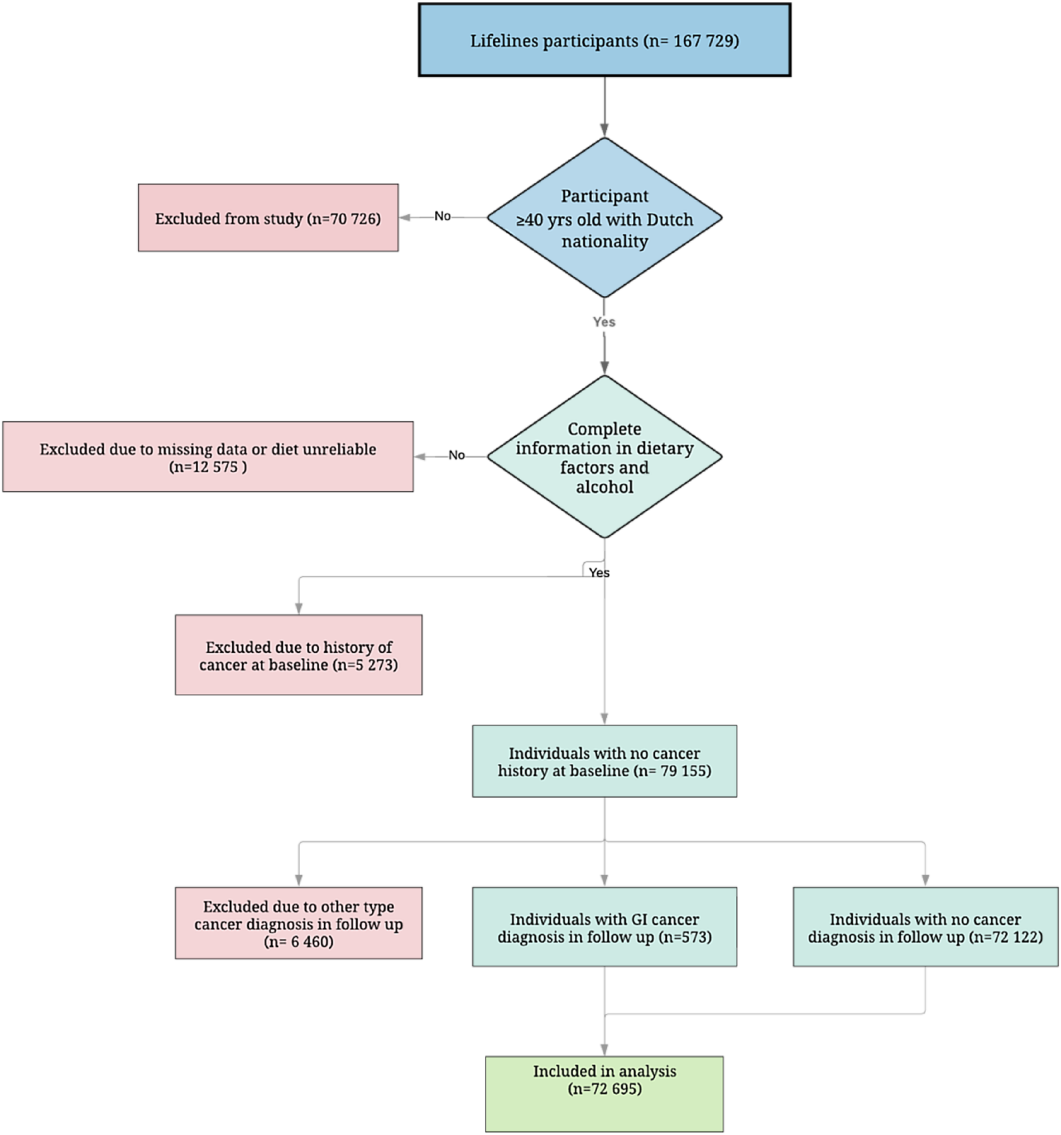
Study flowchart and selection of study population

The following participants from the Lifelines cohort were initially included: those aged ≥ 40 years and with Dutch nationality; those with complete data for dietary questionnaires; and those with complete data confirming no history of cancer at baseline (Fig. 1). Next, participants with a pathologically confirmed diagnosis of GI cancer (i.e., CRC, oesophageal, gastric) during the follow-up period from 2006 to 2020 were included as study cases. The reference population comprised participants with no pathologically confirmed cancer diagnosis at either baseline or during follow-up. Data on the incidence of cancer during follow-up were obtained by linkage to a nationwide network and registry of histo-cytopathology in the Netherlands (The PALGA Foundation). PALGA has benefited from having nationwide coverage since 1991, with all Dutch pathology laboratories being digitally connected to provide a national pathology database. Linkage to data from The PALGA Foundation has been in line with the requisite General Data Protection Regulations since May 2018.

### Lifestyle and anthropometric data collection

Structured and validated self-administered questionnaires were used in the Lifelines study to collect data on demographics, health status, dietary intake, and lifestyle. The questionnaire consisted of dietary intake, psychosocial aspects, health status, lifestyle, and demographics, which was sent in two sections to participants after receiving the consent form. Baseline anthropometric measurements were taken at one of the Lifelines research sites [20]. Anthropometric measurements were performed by an experience nurse in first visit (with 1-h duration). Height and weight were measured without shoes and heavy clothing using SECA222 stadiometer, SECA 761 scale, respectively. Waist and hip circumference were measured by SECA 200 measuring tape. The detailed description on the questionnaires an application procedure is described elsewhere [20].

### Food intake assessment and dietary quality indices

*Food intake* Dietary intake was assessed using a validated Food Frequency Questionnaire (FFQ) with 110 food items that were included by Lifelines [21]. This FFQ is a self-administered semi-quantitative questionnaire which was based on a national survey on food consumption in the Dutch population for 1997. It quantifies individual dietary intake over the month preceding completion of the FFQ. Reported food groups and alcohol intakes were extracted based on the Dutch food composition database [21, 22].

A validation study showed that the applied FFQ provides an accurate estimation of the mean level of energy intakes of the participants, and ranks them accurately according to their intake [21]. Given the inclusion of food groups in scoring system of diet quality indices, the average intake of individual scoring components relevant to four nutrition indices were calculated by multiplying the intake frequencies of a given food (belonging to the included food groups in diet quality indices) by the indicated consumed amounts to obtain intake in grams per day or week. Later, we summed up the values for the food group across all the relevant foods.

*Dietary indices* We used four nutritional indices to quantify dietary quality per individual. The component cut-off points and scoring for the applied dietary indices are summarized in Supplementary Tables 1 and 2. In all four indices, higher scores represent higher diet quality.

*The Dutch Dietary Guidelines (DDG) Index* is based on current international scientific evidence on the impact of food components and dietary habits in the development of ten diet-related chronic diseases in the Netherlands, including CRC [23]. Food components that yield a higher diet quality score in the DDG index include vegetables, fruits, legumes, whole-grain products, unsalted nuts, fish, soft margarine, liquid cooking fats, vegetable oil, and tea. Components yielding a lower dietary quality score include red and processed meat, sugar-sweetened beverages, and alcohol. The DDG index uses a cut-off score for sufficient consumption of the food item introduced by Dutch dietary guidelines or using surveys on food consumption from the Netherlands Nutrition Center. Every participant was scored as adhering (1) or not adhering (0) to the recommended amount per component. The overall DDG score is calculated as the sum of scores, with a theoretical range from 0 (no adherence) to 13 (complete adherence).

*The Lifelines Diet Score (LLDS), similar to DDG,* is based on the 2015 Dutch dietary guidelines, [23, 24]. It is calculated using nine food groups for a high-quality diet, namely vegetables, fruit, whole-grain products, legumes and nuts, fish, oils and soft margarine, unsweetened dairy, filtered coffee, and tea; in addition, three food groups yield a lower quality score, namely red and processed meat, sugar-sweetened beverages, and butter and hard margarine. Intake (g/1000 kcal/food group) is categorized into quintiles, with scores of 0–4 given for the lowest to the highest quintile for the high-quality diet items and reverse scoring (from 4 to 0 for the lowest to the highest quintiles) for the low-quality diet items. The total score for each participant was estimated as the sum of the scores across all food groups (range, 0–46). The development of the LLDS score has been described elsewhere [24].

*The American Cancer Society (ACS) Index*, which is based on recommendations provided by the ACS for cancer prevention, has three main components that concern maintaining a healthy BMI, moderate/intensive level of physical activity, and healthy dietary habits. Food components that improve diet quality consist of fruits, vegetables, and whole grains, while red/processed meat and alcohol downgrade diet quality. The food components (excluding alcohol) were categorized into quartiles from lowest (0) to highest (3) quartiles for the healthier item and scored in reverse from the lowest (3) to the highest (0) quartile for red and processed meat intake. BMI, physical activity, and alcohol intake were categorized based on established criteria [17]. The scores for all components were then added together to give an overall score that ranged from 0 to 15 points per individual.

*The World Cancer Research Fund/American Institute for Cancer Research (WCRF/AICR) Index* [4]*,* defined based on cancer prevention recommendations for 2018, relied on BMI, waist circumference, physical activity, and dietary components. For this, fruits, vegetables, and whole grains are taken to increase diet quality, whereas red/processed meat, energy-dense foods, sugar-sweetened beverages, and alcohol are taken to worsen diet quality. Each component was scored for complete adherence (0.50–1.00), partial adherence (0.25–0.50), and non-adherence (0.00–0.25) to the recommended intake. The overall WCRF/AICR score could range from 0 to 6 points [4].

### Data analysis

Demographic and health-related characteristics were described overall and stratified by the incidence of GI cancer. The resulting scores for each diet quality index were categorized in quintiles. The follow-up time per person was defined as the period from completing the baseline questionnaire to the date of a pathological report of GI cancer, death, loss to follow-up, or last follow-up (to 01-01-2020) (i.e., right censoring), whichever occurred first. For the analysis, GI cancers were classified based on tumor site into CRC and upper GI cancer (esophagus and gastric cancers). The trend in change in CRC and upper GI cancer incidence by an increase in diet quality was tested by calculating *p* for trend by multivariable Cox proportional hazard models, using the diet quality indices as continuous variables.

Multivariable Cox proportional hazard models were applied to quantify the association between diet quality and GI cancer risk, generating hazard ratios (HR) and 95% confidence intervals (95% CI). We calculated the HR separately by tumor site (CRC and upper GI cancers) in each quintile of the diet quality indices. We generated a log–log (survival) versus a log-time plot to assess the risk proportionality assumption, which was not violated.

Models were adjusted for age (continuous; years), height (continuous; cm) [25], family history of cancer (yes, no), educational level (categorical; low, medium, and high), smoking (continuous; pack/year), BMI (continuous; kg/m^2^, [26]), physical activity (continuous; leisure [i.e. cycling, dancing], household [i.e. gardening, home repair], work [i.e. construction, farming], school [i.e. sports lessons], and moderate-and-vigorous activities hrs/wk [i.e. conditioning exercise] [27]), sedentary behavior (continuous; ≥ 2 h/day TV watching, hrs/wk), energy intake (excluding alcohol; continuous; g/d). The scoring system in the LLDS was corrected for daily calorie intake, requiring no further adjustment for this factor. Given the inclusion of BMI and physical activity in the scoring for the ACS and WCRF/AICR indices, no adjustment for these variables or sedentary behavior was performed. Finally, to assess the role of food components on the observed association between GI cancer and high dietary quality defined by the ACS and WCRF/AIRC indices, we excluded non-food components (e.g., BMI, waist circumference, and physical activity) from scoring and included them as adjusting variables before recalculating the HRs for the GI cancers.

Sensitivity analyses were conducted in two different ways: a) by removing the incident cases in the first 2 years of follow-up from the model, as a GI cancer diagnosis might have influenced the diet intake of participants before being diagnosed; b) excluding one food component at a time and checking if a specific food component drove the association between GI cancers and the diet quality indices. Analyses were conducted using IBM SPSS, version 23 (IBM Corp., Armonk, NY, USA).

## Results

Among the participants, there were 573 incident cases of GI cancer (434 CRC and 139 upper GI cancers). These were diagnosed over a median follow-up of 8 years (interquartile range 2 years). The demographic and health-related characteristics of participants are described in Table 1. The mean age of the study population was 51.20 ± 8.71 years at baseline, with incident GI cancers occurring at 58.31 ± 9.66 years. Of note, 42.8% of the study population were men, but these accounted for 60.2% of incident cancers. Also, 37.3% of the study population and 46.6% of those with GI cancers had a low education level.

**Table 1:** The demographic and health-related characteristics of participants in the Lifelines cohort for 2006–2009

Characteristics ^1^	All study population (*n* = 72,695)	Non-cases (*n* = 72,122)	Incident GI cancers (*n* = 573)
Age at inclusion (years)	51.20 (8.71)	51.15 (8.67)	58.31 (9.66)
Sex*			
Women (%)	41 608 (57.2%)	41 380 (57.4%)	228 (39.8%)
Men (%)	31 087 (42.8%)	30 742 (42.6%)	345 (60.2%)
Height (cm)*	174.56 (9.34)	174.55 (9.35)	175.33 (8.98)
Educational level*			
Low	27 220 (37.3%)	26 954 (37.4%)	266 (46.4%)
Medium	26 325 (36.3%)	26 149 (36.3%)	176 (30.7%)
High	19 150 (26.4%)	19 019 (26.3%)	131 (22.9%)
Smoking* (packages/year)	7.42 (10.97)	7.37 (10.91)	12.87 (16.32)
Alcohol consumption* (gr/day)	4.47 (7.88)	4.46 (7.87)	6.34 (9.87)
Calorie intake (Kcal/day)*^2^	2049.21 (602.90)	2049.53 (602.81)	2009.38 (604.03)
BMI (kg/m^2^)*	26.54 (4.21)	26.53 (4.21)	27.46 (4.13)
Physical activity (hrs/wk)	4.22 (4.95)	4.22 (4.94)	4.72 (5.88)
Sedentary Behavior (hrs/wk)*	2.56 (1.50)	2.56 (1.49)	2.87 (1.49)
DDG index (mean score)	5.21 (1.71)	5.21 (1.71)	5.16 (1.76)
LLDS (mean score)*	24.8 (5.92)	24.8 (5.92)	24.8 (6.08)
ACS index (mean score)*	9.28 (2.45)	9.28 (2.45)	9.11 (2.64)
WCRF index (mean score)	3.11 (0.96)	3.11 (0.96)	3.06 (0.94)

Values are presented as means (SDs) or as *n* (%)

*BMI* body mass index; *GI* gastrointestinal; *gr* grams; *kg* kilograms; *hrs* hours; *wk* week

^1^Missing values (*n*, %)

^2^Excluding the calorie intake from alcohol consumption

*Significant difference between non-cases and incident cases (*P* < 0.01

The associations between index-based diet quality and GI cancer are presented in Tables 2 and 3. A significant decreasing trend was detected in CRC risk across categories for the ACS index (*p* = 0.04) when no other significant trend was detected. The highest DDG and LLDS quintiles were not significantly associated with a decreased risk of CRC (HR 0.79; 95% CI 0.57–1.09; HR 0.94; 95% CI 0.68–1.30, respectively) and this remained consistent in the sensitivity analysis (HR 0.72; 95% CI 0.48–1.07; HR 0.91; 95% CI 0.62–1.33, respectively). Similarly, the highest WCRF/AIRC was not significantly associated with a reduced risk of CRC (HR 0.74; 95% CI 0.54–1.01). An increase in diet-lifestyle quality, quantified as the highest quintile of ACS, was associated with a reduced risk of CRC (HR 0.62 95% CI 0.46–0.84), the significant association remained persistent among those diagnosed with GI cancers after 2 years of follow-up (HR 0.57 95% CI 0.39–0.83), see supplementary Table 3. HRs for quintiles of the ACS and WCRF/AIRC indices remained consistent after excluding the non-food components from these scores.

**Table 2:** The association between dietary quality quantified by nutritional indices and CRC risk in the Lifelines cohort for 2006–2020 (*n* = 72,695)

CRC	Quintiles of the scores of dietary indices						
	1	2	3	4	5		*P*_trend_
DDG index							
	624	541	636	626	577		
HR [95% CI]	Ref	0.84 (0.61–1.17)	0.92 (0.68–1.25)	0.90 (0.66–1.23)	0.79 (0.57–1.09)		0.61
LLDS							
	654	716	417	556	630		
HR [95% CI]	Ref	0.87 (0.63–1.20)	0.83 (0.60–1.16)	0.72 (0.52–1.01)	0.94 (0.68–1.30)		0.29
ACS index							
	718	656	514	536	583		
HR [95% CI]	Ref	0.88 (0.65–1.19)	**0.67 (0.49–0.91)**	**0.64 (0.49–0.84)**	**0.62 (0.46–0.84)**		**0.04**
ACS index excluding lifestyle factors							
	718	656	514	536	583		
HR [95% CI]	Ref	0.91 (0.65–1.26)	**0.75 (0.57–0.99)**	**0.65 (0.46–0.92)**	**0.68 (0.49–0.93)**		0.60
WCRF index							
	610	584	619	614	578		
HR [95% CI]	Ref	0.88 (0.65–1.20)	0.90 (0.68–1.21)	0.83 (0.61–1.12)	0.74 (0.54–1.01)	0.39	
WCRF index excluding lifestyle factors							
	610	584	619	614	578		
HR [95% CI]	Ref	0.98 (0.71–1.35)	0.96 (0.70–1.31)	1.01 (0.74–1.38)	0.90 (0.65–1.26)		0.96

Analyses were performed by Cox proportional hazard models and results are presented as HRs [95% CIs]. The adjustment was for age (continuous; years), height (continuous; cm), family history of cancer (yes, no), educational level (categorical; low, medium, and high), smoking (continuous; pack/year), BMI (continuous; kg/m^2)^, physical activity (continuous; leisure, household, work, school, and moderate-and-vigorous activities hrs/wk), sedentary behavior (continuous; ≥ 2 h/day TV watching, hrs/wk), energy intake (excluding alcohol; continuous; g/d). The scoring system in the LLDS was corrected for daily calorie intake, requiring no further adjustment for this factor. Given the inclusion of BMI and physical activity in the scoring for the ACS and WCRF/AICR indices, no adjustment for these variables or sedentary behavior was performed

*ACS* American Cancer Society; *BMI* body mass index; *CI* confidence interval; *CRC* colorectal cancer; *DDG* Dutch dietary guidelines; *HR* hazard ratios; *LLDS* Lifelines Diet Score; *WCRF/AICR* World Cancer Research Fund/American Institute for Cancer Research

**Table 3:** The association between dietary quality quantified by nutritional indices and UGI cancer risk in the Lifelines cohort for 2006–2020 (*n* = 72,695)

Upper GI Cancer	Quintiles of the scores of dietary indices					*P*_trend_
	1	2	3	4	5	
DDG						
	258	157	172	175	213	
HR [95% CI]	Ref	0.60 (0.34–1.05)	0.60 (0.35–1.01)	0.61 (0.36–1.05)	0.71 (0.42–1.20)	0.11
LLDS						
	256	223	184	131	169	
HR [95% CI]	Ref	1.59 (0.90–2.82)	1.36 (0.75–2.48)	0.95 (0.51–1.78)	1.03 (0.54–1.97)	0.20
ACS						
	216	179	248	155	182	
HR [95% CI]	Ref	0.86 (0.49–1.51)	1.18 (0.72–1.94)	0.73 (0.45–1.20)	0.85 (0.50–1.46)	0.52
ACS index excluding lifestyle factors						
	216	179	248	155	182	
HR [95% CI]	Ref	0.91 (0.49–1.67)	1.19 (0.74–1.91)	0.38 (0.17–0.85)	0.94 (0.53–1.66)	0.52
WCRF						
	218	216	186	159	183	
HR [95% CI]	Ref	0.95 (0.57–1.57)	0.83 (0.50–1.37)	0.70 (0.40–1.21)	0.80 (0.47–1.35)	0.71
WCRF index excluding lifestyle factors						
	218	216	186	159	183	
HR [95% CI]	Ref	0.66 (0.39–1.12)	0.61 (0.36–1.03)	0.71 (0.42–1.19)	0.66 (0.38–1.14)	0.36

Analyses were performed by Cox proportional hazard models and results are presented as HRs [95% CIs]. The adjustment was for age (continuous; years), height (continuous; cm), family history of cancer (yes, no), educational level (categorical; low, medium, and high), smoking (continuous; pack/year), BMI (continuous; kg/m^2^), physical activity (continuous; leisure, household, work, school, and moderate-and-vigorous activities hrs/wk), sedentary behavior (continuous; ≥ 2 h/day TV watching, hrs/wk), energy intake (excluding alcohol; continuous; g/d). The scoring system in the LLDS was corrected for daily calorie intake, requiring no further adjustment for this factor. Given the inclusion of BMI and physical activity in the scoring for the ACS and WCRF/AICR indices, no adjustment for these variables or sedentary behavior was performed

*ACS* American Cancer Society; *BMI* body mass index; *CI* confidence interval; *DDG* Dutch dietary guidelines; *GI* gastrointestinal; *HR* hazard ratios; *LLDS* Lifelines Diet Score; *WCRF/AICR* World Cancer Research Fund/American Institute for Cancer Research

No significant trend was detected for changes in the risk of upper GI cancer by an increase in the LLDS and DDG index (*P*_trend_ > 0.05). Similarly, no significant trend was detected for changes in the risk of upper GI cancer by increases in either the ACS index (*P*_trend_ > 0.05) or the WCRF/AICR index (*P*_trend_ > 0.05). Higher quintiles on the DDG and LLDS indices were not associated with the risk of upper GI cancer (HR 0.71; 95% CI 0.42–1.20; HR 1.03; 95% CI 0.54–1.97, respectively). No significant association with upper GI cancer risk was found for higher quintiles compared to the reference first quintile for either the ACS or the WCRF/AICR, supplementary Table 3, and supplementary Table 4.

## Discussion

A high dietary-lifestyle quality index quantified by the ACS were associated with a reduced risk of CRC, whereas, neither WCRF nor the DDG and the LLDS indices affected CRC risk. Moreover, no strong associations were detected between high dietary quality and a reduction in the risk of upper GI cancer. The observed beneficial findings appeared to occur due to synergy among food components included in the WCRF/AICR and ACS indices rather than any single component having a specific effect.

### Diet quality indices and CRC cancer risk

The findings that high dietary quality, as quantified by the WCRF/AICR and ACS indices, lower the risk of CRC are consistent with the findings of our systematic review [14]. In that review, we pooled the findings from 38 studies and reported that there was a 1.2–1.5-times reduced risk of developing CRC when achieving a high diet quality score on the Diet Inflammatory Index (DII) and Mediterranean Diet Score (MDS) [14]. However, other studies have failed to show a significant reduction in CRC risk with high diet quality [2, 3, 6]. This may be because these studies used incomplete data on the included food components, or failed to adjust for some confounders (e.g., history of NSAID use) [2] or had poorly generalizable study populations due to socioeconomic status, being assessed among health professionals [3], or being assessed among people with Lynch syndrome [6].

Despite another study in the Netherlands reported a significant decrease in CRC risk for individuals per unit increase in the DDG index score [28], we observed no significant association. This may be explained by the other study including participants with a mean age that was approximately 13 years higher than in our study. Furthermore, scoring food components in a binary system of adherence or non-adherence to guidelines, as in the DDG index, may have failed to categorize people efficiently with low and high dietary quality scores.

Comparable results were found after excluding lifestyle factors from the ACS and WCRF/AIRC indices. However, we cannot exclude the possibility that this was because of the low variation of lifestyle factors in our cohort. Similar to our findings, a study in France reported that there was a non-significant beneficial role for high WCRF index scores in the prevention of CRC [19]. In this earlier research, a limited number of incident cases (i.e., 118 CRC cases) may have led to insufficient power to detect significant associations, despite the significant variation in lifestyle factors (e.g., adiposity) in their cohort.

### Diet quality indices and upper GI cancer risk

Conflicting with the results of our recent meta-analysis, in which we found a significantly reduced risk of upper GI cancer with higher diet quality [15], we found no major benefits for diet quality on the risk of upper GI cancer. A high dietary quality, as quantified by the DII and MDS, were shown to be associated with a 1.4–1.7-times decreased risk of upper GI cancer. This was most likely due to the low number of incident cases, which will have decreased the power to detect significant associations. In the meta-analysis, however, we did include four observational studies that reported no preventive effect for high diet quality measured either by DII or MDS on the risk of upper GI cancer [2, 7–9]. This discordance may have resulted from a relative paucity of food components in the diet quality calculation in those observational studies, the limited adjustments for confounders specific to upper GI cancer (e.g., gastroesophageal reflux and Helicobacter pylori), and a low number of incident cases. Moreover, these studies had a lack of variation in the composition of the upper GI cancer subgroups, either including all upper GI cancers [2], nasopharyngeal cancer only [9] esophageal cancer only [7], or gastric cancer only [7, 8].

The null finding for the ACS and WCRF/AIRC indices, which conflicts with the suggested role for physical activity [29] and adiposity [30] in the carcinogenesis of upper GI cancer, might be due to similar physical activity and BMI levels in our study population. In addition, the lack of a preventive effect of high-quality diets may be related to the limited numbers of food components assessed in the ACS and WCRF/AIRC indices (see Supplementary Table 1). Indeed, coffee [31] tea [31] dairy [32] and seafood [33] likely have important beneficial roles, whereas artificially sweetened beverages [34], excess calorie intake [35], and dietary fats intake probably have adverse effects on upper GI carcinogenesis. Given these findings, their inclusion might improve the performance of diet quality indices for risk prediction.

### Dietary factors associated with GI cancer prevention

We applied two general diet quality indices based on the 2015 Dutch Dietary Guidelines, namely the DDG and LLDS, ensuring that we collected information over similar food groups specific to the Dutch population. Other regional nutritional indices, such as the Chinese Healthy Food Patterns index [36], the Dietary Guidelines for Americans Adherence Index [37], have also reported the beneficial role of high diet quality on GI cancer risk reduction. For example, high scores on the Chinese Healthy Food Patterns accompanied a 1.4-times reduced risk of gastric cancer within a 9.28-year follow-up period [36]. A similar effect has been reported for esophageal and nasopharyngeal cancer with the Dietary Guidelines for Americans Adherence Index [37]. However, despite some evidence of benefit with high-quality diets quantified by these indices, the results are not consistent [14, 15].

Discrepancies in the strength and significance of associations in research to date likely result from significant variations in several key aspects: the inclusion of dairy products, which have a proven beneficial role in the prevention of CRC, in the scoring system (e.g., in the MDS, LLDS, and DDG indices); being solely based on food (e.g., the LLDS and DDG indices) or a combination of foods and nutrients (e.g., the MDS and DII indices); including lifestyle factors (e.g., the ACS and WCRF/AIRC indices); including only food components with beneficial effects (e.g., the Dietary Approach to Stop Hypertension index [38]); including foods with beneficial and non-beneficial effects (e.g., the DDG, LLDS, DII, and MDS indices); making corrections for calorie intake (e.g., the LLDS); and using different scoring systems for adherence to recommendations. A summary of components and scoring system of commonly applied dietary quality indices is provided in Supplementary Table 2.

Variations in ethnicity, lifestyle, and dietary habits within a study population will further alter susceptibility to GI cancer and will alter the performance of diet quality indices in the prevention of GI cancer in different populations. Hence, to improve the assessment of the effect of diet quality on GI cancer prevention, it is crucial to apply quality indices generated based on food components that have a clear role in preventing GI cancer, tailored to the lifestyle and dietary habits of target populations.

## Conclusion

In this study, we compared four questionnaire indices used to assess diet quality and given the variation in included food components and in the scoring systems, provided knowledge to inform the best-fit diet quality index for use in the Dutch population. We anticipate that these findings can be applied for the prevention of GI cancer in future research. Our study benefits from having a prospective design, relatively large sample size (for CRC) and a precise method for ascertaining cases of GI cancer by pathological confirmation. Moreover, the inclusion of variables as possible confounders will have reduced the likelihood of residual confounding bias. Some limitations affect the confidence in findings, including the short follow-up time, lack of information on confounders specific to upper GI cancer (e.g., gastroesophageal reflux and Helicobacter pylori), and the small number of incident cases of upper GI cancer, which in turn, might have hindered the detection of significant associations of high diet quality and upper GI cancer risk. It is also worthy to notice the applied FFQ was only validated for energy intake in a group of volunteers. Moreover, the validity for the consumption of specific food groups which might be different from the validity of energy intake was not considered in the validation study. Accordingly, substantial variation from actual dietary intake can be expected. In future studies, validation of the dietary intake by nutrient biomarkers in blood may provide more confidence in findings. Though the latter is barely feasible in population-based large studies. As another limitation, given the lack of information on micronutrients and macronutrient intake in the Lifelines database, it was not feasible to assessed diet quality measured by Diet inflammatory index and Mediterranean scores or by the Dutch Healthy Diet score (as a frequently applied diet quality index in Dutch studies) for the study population. Given the fact that the latter dietary indices are calculated based on micronutrient and macronutrient intakes rather than whole foods.

Overall, we conclude that a high diet quality score, as measured by the ACS index , was associated with a significantly reduced risk of CRC. This index may be of use in a colorectal cancer prevention program. However, none of the studied diet quality indices had a major effect on the risk of upper GI cancer. Further research is needed to develop quality indicators that target undesirable dietary and lifestyle habits in homogenous populations, to develop a common tool that is culturally validated into different populations.

## Supplementary Information

Below is the link to the electronic supplementary material.

Supplementary file1 (DOCX 52 KB)
