# Supplementary material for: Diet quality indices and gastrointestinal cancer risk: results from the Lifelines study
Source: Eur J Nutr. 2021 Aug 2;61(1):317–27. doi: 10.1007/s00394-021-02648-3 (PMC8783875; doi:10.1007/s00394-021-02648-3)
Supplement: Supplementary file 1 — Supplementary file1 (DOCX 52 KB) [file 394_2021_2648_MOESM1_ESM.docx]

**Supplemental table 1:** Computation of the DDG index, LLDS, ACS and WCRF/AICR, in Lifelines study.

|  |  | DDG | Appointed scores | LLDS | Appointed scores | ACS | Appointed scores | WCRF/AICR | Appointed scores |
| --- | --- | --- | --- | --- | --- | --- | --- | --- | --- |
| Adiposity | Body mass index | - | - |  | - | 18.5-24.9 kg/m²  25-29.9 kg/m²  30-34.9 kg/m^2^  >35.0 | 3  2  1  0 | 18.5-<25 kg/m²  25-<30 kg/m²  <18.5 or ≥30 kg/m² | 0.5  0.2  0 |
|  | Waist circumference |  |  |  |  |  |  | Men: <94 (<37)  Women: <80 (<31.5)  Men: 94–<102 (37–<40)  Women: 80–<88 (31.5–<35)  Men: ≥102 (≥40)  Women: ≥88 (≥35) | 0.5  0.25  0 |
| Physical activity | Physical activity | - | - | - | - | ≥150min/wk  <150min/wk | 1  0 | ≥150min/wk  75-<150  <75min/wk | 1  0.5  0 |
| Plant based foods | Fruits & vegetables | Vegetables ≥200 g/day  Fruits≥200 g/day  Vegetables <200 g/day  Fruits<200 g/day | 1  1  0  0 | Quintile of reported intake | 0  1  2  3  4 | quartiles | 0  1  2  3 | ≥400g/day  200-<400g/day  <200g/day | 0.5  0.25  0 |
|  | Whole grain (7gr fiber per 100 gr) | - | - | Quintile | 0  1  2  3  4 | - | - | ≥400g/day  200-<400g/day  <200g/day | 0.5  0.25  0 |
|  | Legumes | Legumes ≥135 g/week  Legumes <135 g/week | 1  0 | Quintile | 0  1  2  3  4 | - | - | - | - |
|  | Whole grain food | Whole grain products ≥90 g/day  Whole grain products <90 g/day | 1  0 | - | - | Quartile of the ratio of whole grain to refined grains | 0  1  2  3 | - | - |
|  | Nuts | Nuts ≥15 g/day  Nuts <15 g/day | 1  0 | Quintile | 0  1  2  3  4 | - | - | - | - |
|  | Bread, cereals, potatoes  and legumes | Whole grains ≥50% of total grains  Whole grains <50% of total grains | 1  0 |  |  | - |  | - | - |
| Fish | - | Fish ≥100 g/week  Fish <100 g/week | 1  0 | Quintile | 0  1  2  3  4 | - | - | - | - |
| Animal foods ^c^ | Meat and meat products | Red and processed meat<300 g/week  Red and processed meat≥300 g/week | 1  0 | Quintile | 4  3  2  1  0 | Quartile of red and processed meat | 3  2  1  0 | Red/processed meat <500g/week and processed meat <3 g/day  Red/processed meat <500g/week and processed meat 3-<50 g/day  Red/processed meat ≥500g/week or processed meat ≥50 g/day | 1  0.5  0 |
|  | Dairies unsweetened | Dairy ≥350 g/day  Dairy <350 g/day | 1  0 | Quintile | 0  1  2  3  4 | - | - | - | - |
|  |  |  |  | Quintile |  |  |  |  |  |

|  |  | **DDG** | Appointed scores | **LLDS** | Appointed scores | **ACS** | Appointed scores | **WCRF/AICR** | Appointed scores |
| --- | --- | --- | --- | --- | --- | --- | --- | --- | --- |
| **Food that promote weight gain** | **Energy density** | **-** |  |  |  | - |  | **-Teritile of fast foods** | -1  0.5  0 |
|  | **Sugary drinks** | Sugar-containing beverages ≤150 mL/day  Sugar-containing beverages >150 mL/day | 1  0 | quintile | 4  3  2  1  0 | - | - | **Total sugar-sweetened drinks (g**/**day):**  0  >0–_250  >250 | -  1  0.5  0 |
|  | **Sweetened foods** |  |  |  |  |  |  |  |  |
| **Alcohol** |  | Alcohol ≤10 g/day  Alcohol >10 g/day | 1  0 | quintile | 4  3  2  1  0 | ≤3 drink a day (♂) or ≤2 drink a day (♀)    >non (♂) or non (♀)  >1-2 drink a day (♂) or 1drink a day(♀) | 0  1  2 | 0  0-≤28g/day (♂) or ≤14g (♀)  >28 (♂) or >14 (♀) | 1  0.50  0 |
|  | **-** | - | - | - | - | - | **-** | - | - |
| **Oils and fates** | **-** | Unsaturated fats and oils ≥50% of total fats  Unsaturated fats and oils <50% of total fats | 1  0 | Oil and soft margarine (quintile)  Butter hard margarine (quintile) | 0  1  2  3  4  4  3  2  1  0 | - | - | - | - |
| **Tea** | **-** | Tea ≥450 mL/day  Tea <450 mL/day | 1  0 | quintile | 0  1  2  3  4 | - | - | - | - |
| **Coffee** | **-** | - | - | quintile | 0  1  2  3  4 | - | - | - | - |

Abbreviation: ACS: American Cancer Society , DDG: Dutch dietary guidelines, LLDs: Lifelines Diet Score, WCRF/AICR: World Cancer Research Fund/American Institute for Cancer Research.

^a^ LLDs, ACS and WCRF/AIRC used categorical scoring.

^b^ The highest category corresponds to participants who practiced vigorous-intensity activity on at least 3 days during the week and accumulating at least 1500 MET-minutes/week, or 7 days of any combination of walking, moderate-intensity or vigorous intensity activities achieving a minimum of at least 3000 MET-minutes/week. The category of moderate physical activity corresponds to any one of the following 3 criteria: 3 or more days of vigorous activity of at least 20 minutes per day or 5 or more days of moderate-intensity activity or walking of at least 30 minutes per day, or 5 or more days of any combination of walking, moderate-intensity or vigorous intensity activities achieving a minimum of at least 600 MET-minutes/week. The lowest category concerns participants with the lowest level of physical activity, who did not meet criteria of the categories “high” or “moderate”.

^e^ Number of soft drinks servings

**Supplementary Table 2**: Included component and categorization for level of adherence to dietary recommendations in scoring system of commonly applied dietary quality indices^1^

| Dietary quality indices | Investigated in the present study | | | | | | | Other diet indices – investigated in our previous meta-analysis ^1, 2^ | | | | | |
| --- | --- | --- | --- | --- | --- | --- | --- | --- | --- | --- | --- | --- | --- |
|  | DDG | | LLDS | | ACS | | WCRF | DII | MDS | | HEI | | DASH |
| Scoring Components with beneficiary health effects | | | | | | | | | | | | | |
|  | | | | | | | | | | | | | |
| Scoring categorization | Binary categorization | Quintile | | Quartiles | | Tertiles | | Varies between tertiles to quintiles | Varies between tertiles to quintiles | Varies between tertiles to quintiles | | Varies between tertiles to quintiles | |
| Physical activity and adiposity factors | 🗶 | 🗶 | | ✓ | | ✓ | | 🗶 | 🗶 | 🗶 | | 🗶 | |
| Plant based foods^2^ | ✓ | ✓ | | ✓ | | ✓ | | ✓ | ✓ | ✓ | | ✓ | |
| Dairies | ✓ | ✓ | | 🗶 | | 🗶 | | 🗶 | ✓ | 🗶 | | 🗶 | |
| Sea foods | ✓ | ✓ | | 🗶 | | 🗶 | | 🗶 | ✓ | 🗶 | | ✓ | |
| Tea and coffee | ✓ | ✓ | | 🗶 | | 🗶 | | 🗶 | 🗶 | 🗶 | | 🗶 | |
| Oils and Soft margarines | ✓ | ✓ | | 🗶 | | 🗶 | | ✓ | ✓ | ✓ | | ✓ | |
| Scoring Components with undesired health effects | | | | | | | | | | | | | |
| Red and processed meat | ✓ | ✓ | | ✓ | | ✓ | | ✓ | ✓ | 🗶 | | 🗶 | |
| Calorie dense foods^3^ | ✓ | ✓ | | ✓ | | ✓ | | 🗶 | ✓ | ✓ | | 🗶 | |
| Saturated fats | ✓ | ✓ | | 🗶 | | 🗶 | | ✓ | ✓ | ✓ | | ✓ | |
| Adjustments for calorie intake | 🗶 | ✓ | | 🗶 | | 🗶 | | 🗶 | 🗶 | 🗶 | | 🗶 | |

^1^ ‘✓; “The component is considered in scoring system”, ‘🗶’; “The component is not considered in scoring system”.

^2.^ Including fruits, vegetables, whole grains, pulses and legumes.

^3.^Including sweetened beverages, fast foods, foods with added sugar.

**Abbreviation**: ACS: American Cancer Society, DASH; Dietary Approach to Prevent Hypertension, DDG: Dutch dietary guidelines, DII; Diet Inflammatory Index, HEI; Healthy Eating Index, LLDS: Lifelines Diet Score, MDS; Mediterranean Diet Score, WCRF/AICR: World Cancer Research Fund/American Institute for Cancer Research.

^a^ LLDS, ACS and WCRF/AIRC used categorical scoring

**Supplementary table 3.** Sensitivity analysis excluding the cancer diagnosis on the first two years of follow up for the association between dietary quality quantified by nutritional indices and CRC risk in the Lifelines cohort for 2006–2020 (n = 72,695)

| CRC | Quintiles of the scores of dietary indices | | | | | | | | | | | | |
| --- | --- | --- | --- | --- | --- | --- | --- | --- | --- | --- | --- | --- | --- |
|  | 1 | 2 | 3 | 4 | 5 | | | | P _trend_ | | | | |
| **DDG index** | | | | | | | | | | | | | |
| Incidence | 50 | 59 | 79 | 64 | | 59 | | |  | | | | |
| HR [95% CI] | Ref HR = 1 | 0.97 (0.66–1.41) | 0.97 (0.67–1.38) | 0.87 (0.60–1.27) | | | 0.72 (0.48–1.07) | | | | 0.42 | | |
| **LLDS** | | | | | | | | | | | | | |
| Incidence | 50 | 60 | 60 | 64 | | | 77 | | |  | | | |
| HR [95% CI] | ref | 0.79 (0.54–1.16) | 0.85 (0.58–1.24) | 0.72 (0.49–1.06) | | | 0.91 (0.62–1.33) | | | | | 0.46 | |
| **ACS index** | | | | | | | | | | | | | |
| Incidence | 99 | 49 | 33 | 77 | | | 53 | | |  | | | |
| HR [95% CI] | ref | **0.83 (0.59–1.18)** | **0.49 (0.33–0.73)** | **0.61 (0.44–0.84)** | | | **0.57 (0.39–0.83)** | | | | | | **0.002** |
| **WCRF index** | | | | | | | | | | | | | |
| Incidence | 72 | 52 | 66 | 60 | | | | 61 |  | | | | |
| HR [95% CI] | ref | 0.83 (0.58–1.19) | 0.85 (0.60–1.19) | 0.78 (0.54–1.16) | | | **0.69 (0.48–0.99)** | | | | | 0.38 | |

Analyses were performed by Cox proportional hazard models and results are presented as HRs [95% CIs]. Adjustment was for age (continuous; years), height (continuous; cm), family history of cancer (yes, no), educational level (categorical; low, medium, and high), smoking (continuous; pack/year), BMI (continuous; kg/m^2)^, physical activity (continuous; leisure, household, work, school, and moderate-and-vigorous activities hrs/wk), sedentary behavior (continuous; ≥2 hours/day TV watching, hrs/wk), energy intake (excluding alcohol; continuous; g/d). The scoring system in the LLDS was corrected for daily calorie intake, requiring no further adjustment for this factor. Given the inclusion of BMI and physical activity in the scoring for the ACS and WCRF/AICR indices, no adjustment for these variables or for sedentary behavior were performed. **Abbreviations:** ACS, American Cancer Society; BMI, body mass index; CI, confidence interval; CRC, colorectal cancer; DDG, Dutch dietary guidelines; HR, hazard ratios; LLDS, Lifelines Diet Score; WCRF/AICR, World Cancer Research Fund/American Institute for Cancer Research.

**Supplementary table 4.** Sensitivity analysis excluding the cancer diagnosis on the first two years of follow up for the association between dietary quality quantified by nutritional indices and UGI cancer risk in the Lifelines cohort for 2006–2020 (n = 72,695)

| Upper GI Cancer | Quintiles of the scores of dietary indices | | | | | | | | | | | |
| --- | --- | --- | --- | --- | --- | --- | --- | --- | --- | --- | --- | --- |
|  | 1 | 2 | | 3 | 4 | 5 | P _trend_ | | | | | |
| **DDG** | | | | | | | | | | | | |
| Incidence | 22 | | 15 | 19 | 17 | 27 |  | | | | | |
| HR [95% CI] | Ref HR = 1 | | 0.56 (0.29–1.09) | 0.52 (0.28–0.98) | 0.53 (0.28–1.01) | 0.78 (0.43–1.41) | | 0.17 | | | | |
| **LLDS** | | | | | | | | | | | | |
| Incidence | 14 | 24 | | 25 | 18 | 19 | | |  | | | |
| HR [95% CI] | ref | 1.19 (0.61–2.32) | | 1.38 (0.71–2.68) | 0.84 (0.41–1.71) | 1.01 (0.49–2.01) | | | 0.55 | | | |
| **ACS** | | | | | | | | | | | | |
| Incidence | 26 | 12 | | 22 | 19 | 21 | | |  | | |  |
| HR [95% CI] | ref | 0.83 (0.41–1.66) | | 1.37 (0.76–2.46) | 0.67 (0.36–1.26) | 1.10 (0.57–2.11) | | | 0.22 | | | |
| **WCRF** | | | | | | | | | | | | |
| Incidence | 24 | 22 | | 18 | 16 | 20 | | | | |  | |
| HR [95% CI] | ref | 1.10 (0.61–1.97) | | 0.75 (0.40–1.40) | 0.71 (0.37–1.37) | 0.81 (0.52–1.52) | | | | 0.63 | | |

Analyses were performed by Cox proportional hazard models and results are presented as HRs [95% CIs]. Adjustment was for age (continuous; years), height (continuous; cm), family history of cancer (yes, no), educational level (categorical; low, medium, and high), smoking (continuous; pack/year), BMI (continuous; kg/m2), physical activity (continuous; leisure, household, work, school, and moderate-and-vigorous activities hrs/wk), sedentary behavior (continuous; ≥2 hours/day TV watching, hrs/wk), energy intake (excluding alcohol; continuous; g/d). The scoring system in the LLDS was corrected for daily calorie intake, requiring no further adjustment for this factor. Given the inclusion of BMI and physical activity in the scoring for the ACS and WCRF/AICR indices, no adjustment for these variables or for sedentary behavior were performed. Abbreviations: ACS, American Cancer Society; BMI, body mass index; CI, confidence interval; DDG, Dutch dietary guidelines; GI, gastrointestinal; HR, hazard ratios; LLDS, Lifelines Diet Score; WCRF/AICR, World Cancer Research Fund/American Institute for Cancer Research.
